# Biocontrol potential of *Trichoderma*-derived chitinase: optimization, purification, and antifungal activity against soilborne pathogens of apple

**DOI:** 10.3389/ffunb.2025.1618728

**Published:** 2025-07-24

**Authors:** Akanksha, Satish K. Sharma, Bhupesh K. Gupta, Neerja Rana, Anju Sharma, Pramod Verma

**Affiliations:** Department of Plant Pathology, Dr. Yashwant Singh Parmar University of Horticulture and Forestry, Solan, Himachal Pradesh, India

**Keywords:** biological control, chitin, chitinase, soil borne, *Trichoderma*

## Abstract

Apple is most important fruit crop in Himachal Pradesh, contributing substantially to the state’s economy. However, soilborne diseases have emerged as a major concern affecting nursery-raised apples. *Trichoderma* species produce chitinase, an enzyme that degrades chitin, a major component of the fungal cell wall. This study aimed to optimize the growth parameters for chitinase production, extraction, purification, and characterization and to assess the antifungal potential against soilborne pathogens of apple. A total of 14 isolates of *Trichoderma* spp. produced chitinases in a colloidal chitin agar (CCA) medium to varying extents. The optimal incubation period, pH, substrate concentration, and incubation temperature were 7 days, 5, 1%, and 30°C, respectively, while the thermal and pH stability ranged from 30°C to 50°C and from 4 to 6, respectively. Chitinases were purified from *Trichoderma atroviride* UHFTA005 and UHFTA006 and from *Trichoderma virens* UHFTV017 with a molecular mass of 40 kDa. The chitinase from *T*. *atroviride* UHFTA005 at 0.60 μl inhibited the *in vitro* growth of *Dematophora necatrix* (92.22%) and *Sclerotium rolfsii* (91.11%). In a further *in vivo* evaluation of the chitinases, *T*. *atroviride* UHFTA005 was found to be more effective against white root rot and seedling blight of apple, with disease control of 86.67% and 73.33%, respectively, and with 86.67% white root rot disease control in nursery field conditions suggesting its strong potential as a biocontrol agent in nursery field conditions.

## 1 Introduction

Apple (*Malus* × *domestica*) is a deciduous tree in the Rosaceae family. It is one of the world’s most well-known fruits, with an annual production of more than 80 million tonnes (http://faostat.fao.org). It is also one of the most profitable fruit types in terms of foreign exchange. Long-term continuous cropping can disrupt the microbial community, reducing the number of beneficial microbes while increasing the number of soilborne diseases (Chadfield et al., 2022). Apple production is under threat due to global diseases, particularly those produced by soilborne pathogens due to their persistence in the soil and the limited effectiveness of chemical treatments. Climate change hampers management by shifting the soil temperature and moisture levels, promoting pathogen survival and infection.

*Rosellinia necatrix* (Pliego et al., 2012), *Phytopythium cactorum* (Zhou et al., 2022), *Sclerotium rolfsii* (Corazza et al., 1999), *Armillaria mellea* (Marsh, 1952), *Helicobasidium mompa* (Lee et al., 2022), and *Macrophomina phaseolina*, among others, cause major economic losses in agriculture production (Shafique et al., 2016; Surendra et al., 2025). Preventive techniques against soilborne pathogens are needed. The detection of soilborne pathogens is difficult at the early stage; as a result, once infected, plants could not survive (Singh et al., 2019). Traditionally, agrochemicals have been used for their management, but continuous and long-term use has led to soil and water pollution and microbial imbalance, which could result in the emergence of new pathogenic variants (Zhou et al., 2024). The overuse of fungicides has resulted in resistance to some diseases (Jamar et al., 2010).

A more economical and sustainable option to reduce crop losses is “biological control,” in which beneficial microbes are used for disease control (Velivelli et al., 2014). *Trichoderma* spp., *Bacillus* spp., *Pseudomonas* spp., and *Streptomyces* spp. are among the most promising biological control agents for plant disease management (Mukhopadhyay and Kumar, 2020). Apple rhizosphere microorganisms with biocontrol ability can be used against soilborne pathogens while maintaining microbial balance (Velivelli et al., 2014; Zang et al., 2021). *Trichoderma* is widespread, grows quickly, and is easily identified by its enormous number of green conidia. *Trichoderma*, a greenish filamentous fungus, was first reported by Persoon (1794). The morphological characteristics of *Trichoderma* include the presence of hyphae, conidiophores, and phialides (Tulasne and Tulasne, 1865). It is classified under the order Hypocreales, the family Hypocreaceae, and the genus *Trichoderma*. This genus includes saprophytic fungi that are widespread, mycoparasitic, and are beneficial to improving the soil microbial diversity (Mukherjee et al., 2013; Ribeiro dos Santos and Lima dos Santos, 2025).

*Trichoderma* exhibits different mechanisms of action, e.g., competition, hyperparasitism, direct antagonism, secondary metabolite production, and cell wall lysis through the production of different enzymes, including chitinase, glucanase, proteases, and cellulases (Wonglom et al., 2020). *Trichoderma* exhibits mycoparasitism on many pathogens, such as *Pythium*, *Phytophthora*, *Sclerotium*, and *Dematophora*, among others (Shaw et al., 2016). Mycoparasitism is caused by *Trichoderma*-produced secondary metabolites and cell wall disintegrating enzymes (CWDEs). *Trichoderma* species coil around the hyphae of the target fungal pathogens and enter the cell wall by producing CWDEs (Gajera et al., 2012). CWDEs target the pathogen’s cell wall components (β-1,3-glucan and chitin) and utilize them as nutrients, promoting *Trichoderma* growth (Viterbo et al., 2002).

*Trichoderma* species, in addition to controlling plant diseases, improve plant heath, increase plant growth, provide better nutrient utilization, and increase plant resistance. Among all the enzymes, chitinase is of upmost importance as most of the fungal plant pathogenic species inherit chitin in their cell wall as an imperative constituent (Baek et al., 1999; Ulhoa and Peberdy, 1991). Furthermore, *Trichoderma* species produce enzymes that target certain plant diseases (Bastakoti et al., 2017) and have the ability to hydrolyze proteins, cellulose, hemicellulose, and chitin, which directly limit plant diseases (Sharma et al., 2019).

Chitin is a polymer of *N*-acetylglucosamine (NAG), which is the second most abundant polymer after cellulose and is considered as a major component of the fungal cell wall (Haran et al., 1996). The cell wall is mainly composed of Chytridiomycota, Ascomycota, Basidiomycota, and the exoskeleton of insects (Flach et al., 1992). It strengthens the cell wall of fungi and the exoskeleton of insects (Elieh-Ali-Komi and Hamblin, 2016). Chitinase hydrolyzes the β-1,4-glycosidic bond between the NAG residues of chitin (Kitamura and Kamei, 2003). The molecular weights of chitinases range from 20 to 90 kDa. The *Trichoderma* spp. chitinases belong to family 18 of glycosyl hydrolase and are further classified into class III and class V. There are other microorganisms that show chitinolytic activity, including *Bacillus* spp., *Serratia* spp., and *Streptomyces* spp (Dahiya et al., 2006). As chitin is not easily soluble in water, it is chemically altered to create colloidal chitin, which can more easily form a homogeneous distribution on agar medium due to its small particle size (Lingappa and Lockwood, 1962). Therefore, it is one of the effective methods for the prevention and control of soilborne pathogens that can effectively resist chemical pesticides.

In this study, *Trichoderma* spp. were isolated from the rhizospheric region of apple for the extraction of chitinase. The present study also focused on the optimization of the growth parameters for chitinase production, extraction, purification, and characterization and on the assessment of the antifungal potential against soilborne pathogens of apple.

## 2 Materials and methods

### 2.1 Study area

**Figure 1:**
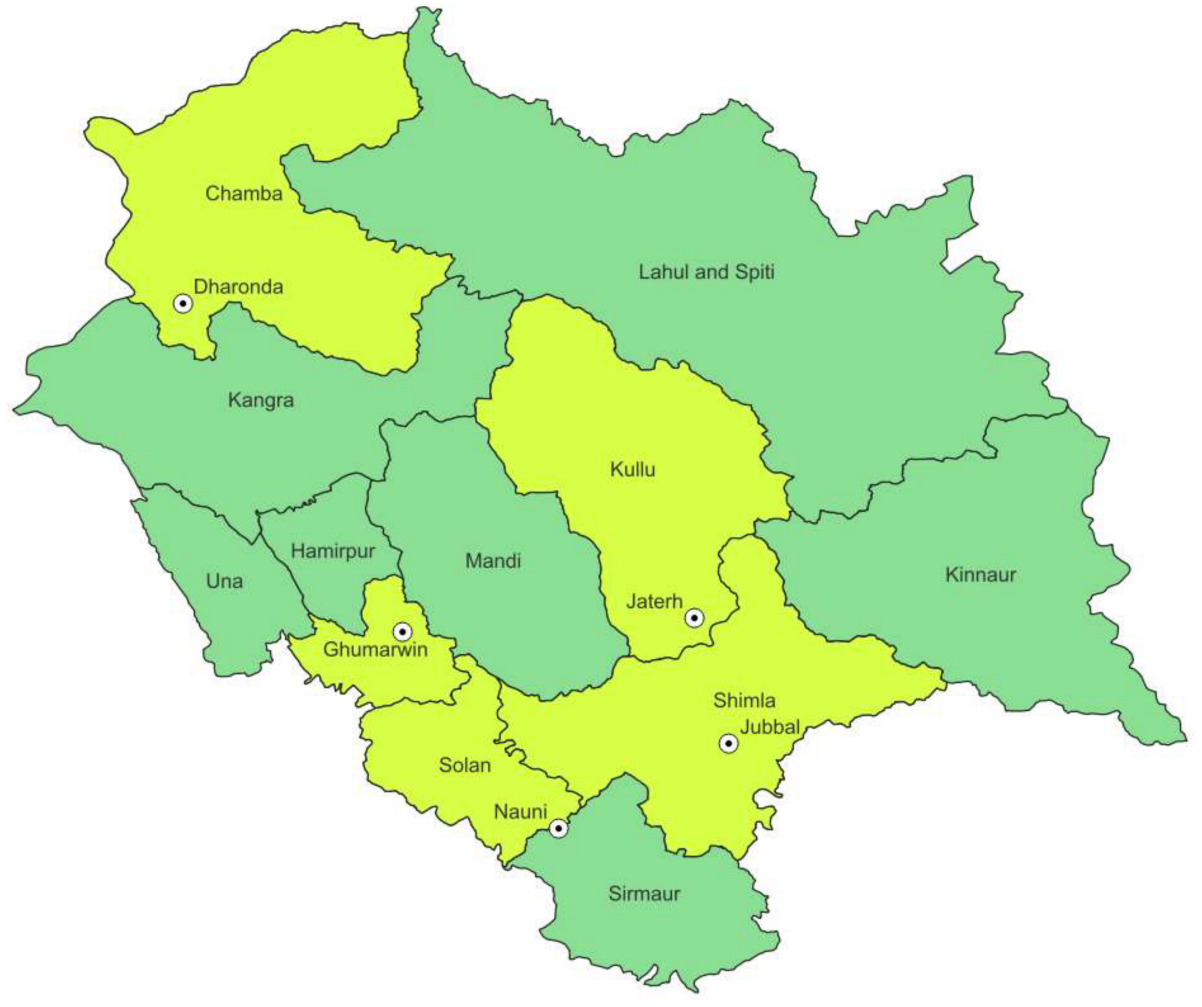
Location map of apple growing areas of Himachal Pradesh showing sampling sites.

A survey was conducted across various prominent apple-growing areas in Himachal Pradesh, in particular Bilaspur, Mandi, Kullu, Solan, and Shimla (**Figure 1**). At each selected site, four sub-sites were selected in orchards/nurseries. Soil samples were collected from the rhizospheric area of apple at 10- to 15-cm soil depth into polythene bags (Singh et al., 2003).

### 2.2 Trichoderma

#### 2.2.1 Isolation of resident *Trichoderma* species

For isolation, the serial dilution technique was followed at 10^5^ dilution for each sample. Of each sample, 100 μl was pipetted out into a plate with potato dextrose agar (PDA) medium and incubated at 30°C for 7 days. Isolate UHFTB002 was procured from the Department of Plant Pathology, Dr Yashwant Singh Parmar University of Horticulture and Forestry Nauni, Solan, Himachal Pradesh.

#### 2.2.2 Isolation of soilborne pathogens of apple

*Dematophora necatrix* was isolated from the roots of the affected seedlings/plants following standard procedure, while *S. rolfsii* was procured from the Department of Plant Pathology, Dr. Yashwant Singh Parmar University of Horticulture and Forestry Nauni, Solan, Himachal Pradesh.

#### 2.3 Genomic DNA extraction and sequencing

Genomic DNA was extracted using the CTAB method (Sambrook and Fritsch, Maniatis, 1989). PCR amplification was carried out in a thermal cycler. ITS1 (5′ TCC GTA GGT GAA CCT TGC GG 3′) and ITS4 (5′ TCC TCC GCT TAT TGA TAT GC 3′) primers were used during PCR amplification. The PCR conditions were as follows: 95°C for 5 min (initial denaturation), 95°C for 1 min (denaturation), 60°C for 1 min (annealing), 72°C for 1 min (extension), and 72°C for 10 min (final extension) with 35 cycles. The PCR product was electrophoresed at 1% agarose gel for 1 h at 100 V and visualized under Gel Doc. The 600-bp PCR product was sent to Eurofins Genomics India Pvt. Ltd., Bengaluru, Karnataka. Sequenced data were analyzed with BLAST (Basic Local Alignment Search Tool; www.ncbi.nlm.nih.gov).

#### 2.4 Phylogenetic analysis

For phylogenetic analysis, the downloaded sequences of *Trichoderma* spp. were aligned using ClustalW with the neighborhood joining method (Thompson et al., 1994) and were edited using BioEdit. Alignment was trimmed using MEGA 11 software (Tamura et al., 2013).

### 2.5 Medium and culture conditions

CCA medium (in grams per liter): MgSo_4_·7H_2_0, 0.3; (NH_4_)_2_·SO_4_, 3.0; KH_2_PO_4_, 2.0; citric acid, 1.0; agar powder, 15; Tween-80, 200 μl; colloidal chitin, 4.5; and bromocresol purple, 0.15. pH was set to 4.7.

PDA medium (in grams per liter): potato extract, 4; dextrose, 20; and agar, 20. The final pH was adjusted to 5.6. This was used for preservation of the fungal cultures.

### 2.6 Preparation of colloidal chitin

Colloidal chitin was prepared from chitin powder. Of the chitin powder, 10 g was treated with 40–50 ml (37%) of HCl. In a 1,000-ml beaker, HCl was slowly added into the chitin powder, with continuous stirring until becoming a slurry. The chitin–HCl mixture was then kept in a refrigerator (4°C) for 1 h incubation. The mixture was treated with 50% (pre-chilled) ethanol and kept in the refrigerator overnight to facilitate better precipitation of colloidal chitin. This mixture was filtered through Whatman no. 1 filter and washed with 1–2 L of pre-chilled distilled water. The pH was set between 6 and 7 by adding NaOH/HCl and was kept in shade after filtration for drying. The ground powder was kept at 4°C for further use.

### 2.7 Preparation of colloidal chitin agar medium for chitinase-producing isolates

The modified protocol of Gomez Ramirez et al. (2004) was used to prepare the CCA mixture, which was then autoclaved at 121°C for 20 min. The sterilized medium was then poured into Petri plates. After solidification of the medium, different isolates of *Trichoderma* were kept at the center of the plate to examine the chitinase-producing isolates with three replications each and incubated at 30°C for 3 days.

### 2.8 Optimization of the growth parameters on chitinase activity and stability

The effects of different growth parameters, such as the incubation period, pH, substrate concentration, and incubation temperature, on chitinase activity were examined. The effect of pH was assessed between 3 and 8 at 1-unit intervals with NaOH/HCl. The effects of five temperature values ranging from 20°C to 40°C with a variation of 5°C and five concentrations of colloidal chitin, i.e., 0.5%, 1%, 1.5%, 2%, and 2.5%, were evaluated for 9 days. The enzyme activity was measured (Miller, 1959).

### 2.9 Enzyme activity assay

The enzyme activity assay was performed according to the method of Miller (1959). The reaction mixture [1 ml crude enzyme solution + 1 ml colloidal chitin (1%) + 1 ml of 0.1 M citrate buffer (pH 6.0)] was incubated in a water bath at 37°C for 30 min. The reaction was stopped by adding 2 ml of a dinitrosalicylic acid (DNS) reagent and incubated in a water bath for 5 min at 60°C for color development. The reaction mixture without crude enzyme was used as blank. The optical density (OD) value was measured at 540 nm using the standard curve of *N*-acetyl glucosamine unit (100–600 ppm). One unit (U) of chitinase activity was defined as the amount of enzyme that released micromoles of NAG per minute per milliliter, while specific activity (SA) represents the micromoles of NAG released per minute per milligram of protein.

### 2.10 Protein estimation using the Bradford method

The concentration of protein was estimated according to Bradford (1976) using Coomassie Brilliant Blue G-250. The OD value was measured at 595 nm using the standard curve of bovine serum albumin (BSA).

### 2.11 Enzyme extraction

Colloidal chitin broth medium with a pH of 5 was prepared in 500-ml flasks, and four to five pieces of 5 mm size were inoculated in each flask for the different isolates of *Trichoderma* and were incubated at 30°C for 7 days. After 7 days, the flasks were kept on a rotary shaker for 1 day and the broth centrifuged at 10,000 rpm for 20 min. The supernatant was collected and then stored in a refrigerator for purification. The supernatant was used as a crude enzyme.

### 2.12 Partial purification

Purification of the crude enzyme was performed with the ammonium sulfate precipitation method (Xu et al., 2014). Ammonium sulfate precipitation was conducted using 0%–45% concentration of ammonium sulfate under 4°C. The precipitates were collected by centrifugation at 10,000 rpm for 20 min and the supernatant was discarded. The pellet was dissolved in citrate buffer (pH 6). Ammonium sulfate traces were removed through dialysis. Sephadex G-100 beads were dissolved in distilled water (10 g of Sephadex G-100 was added into 500 ml of distilled water), which was then poured over the top of the silica and washed with a large amount of citrate buffer for the smooth downward flow of protein. The dialyzed fraction was suspended in a Sephadex G-100 chromatographic column, and the fraction showing maximum chitinase activity was pooled (Laemmli, 1970).

### 2.13 Sodium dodecyl sulfate polyacrylamide gel electrophoresis

The molecular weight of the purified protein was determined using SDS-PAGE. The purified fraction was submitted into 12% SDS-PAGE (Laemmli, 1970), and 40 μg of the sample was loaded and electrophoresis carried out with 150 V of current for approximately 1–2 h. The gel was carefully removed and then placed into a staining solution (Coomassie Brilliant Blue G-250) (Candiano et al., 2004). Thereafter, the staining solution was poured off and the gel was kept in a destaining solution for 3–4 h with continuous shaking for band visualization.

### 2.14 Statistical analysis

Statistical analysis was performed with R software (R Development Core Team, 2008). Experiments were conducted using a completely randomized design (CRD) in triplicates and analyzed using one-way ANOVA. Qualitative analysis of chitinase was performed using Duncan’s multiple range test (DMRT). A *p*-value <0.05 was considered statistically significant.

## 3 Results and discussion

### 3.1 Phylogenetic analysis of isolates of *Trichoderma* species

**Figure 2:**
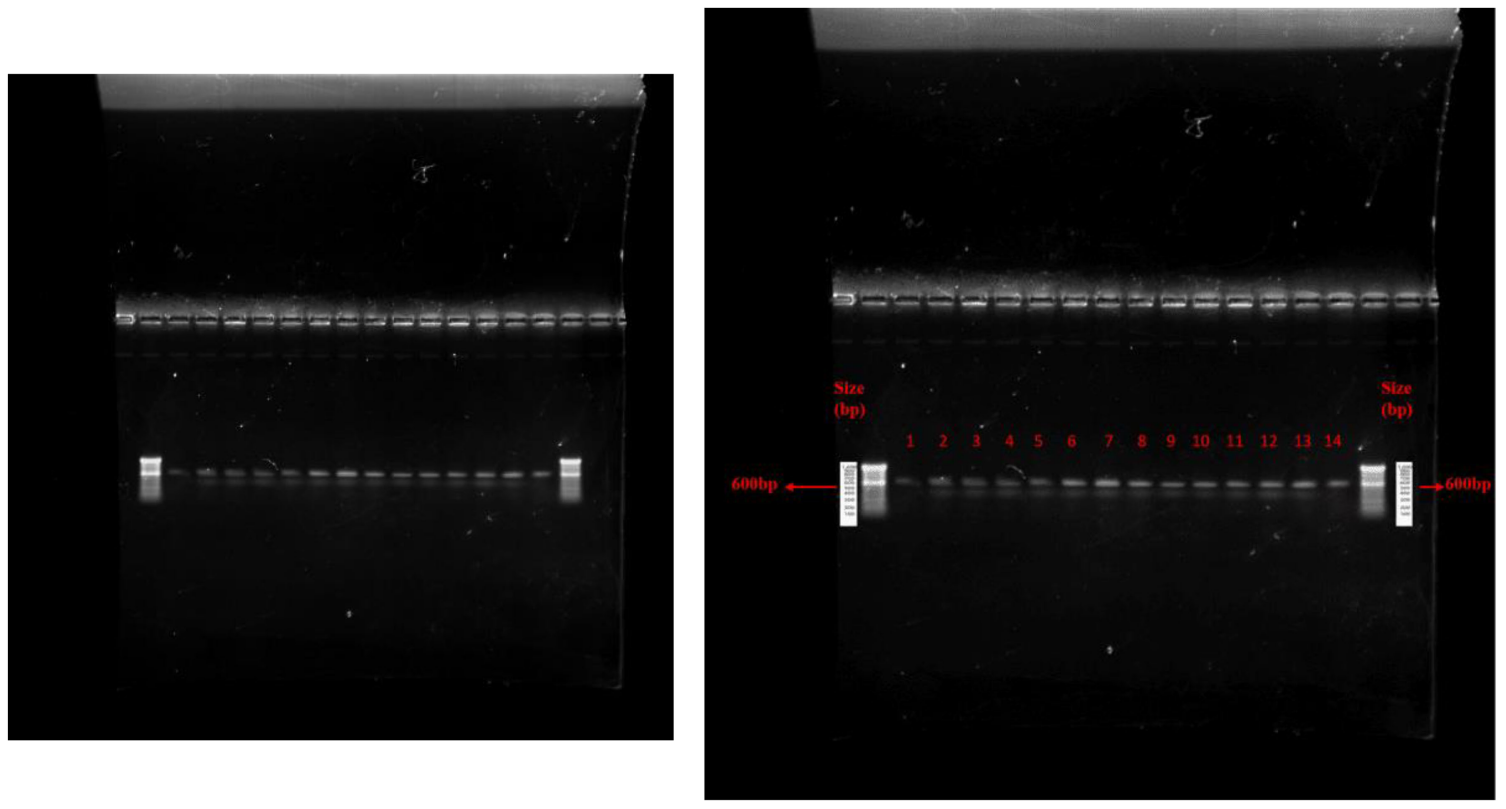
Agarose gel electrophoresis of PCR amplicon from fungal DNA. Where, 1= *Trichoderma atroviride* UHFTA001, 2= *Trichoderma atroviride* UHFTA002, 3= *Trichoderma atroviride* UHFTA003, 4= *Trichoderma atroviride* UHFTA004, 5= *Trichoderma atroviride* UHFTA005, 6= *Trichoderma atroviride* UHFTA006, 7= *Trichoderma atroviride* UHFTA007, 8= *Trichoderma virens* UHFTV017, 9= *Trichoderma harzianum* UHFTH0013, 10= *Trichoderma longibrachiatum* UHFTS001, 11= *Trichoderma virens* UHFTC001, 12= *Trichoderma longibrachiatum* UHFTK001, 13= *Trichoderma brevicompactum* UHFTB001, 14= *Dematophora necatrix*.

**Figure 3:**
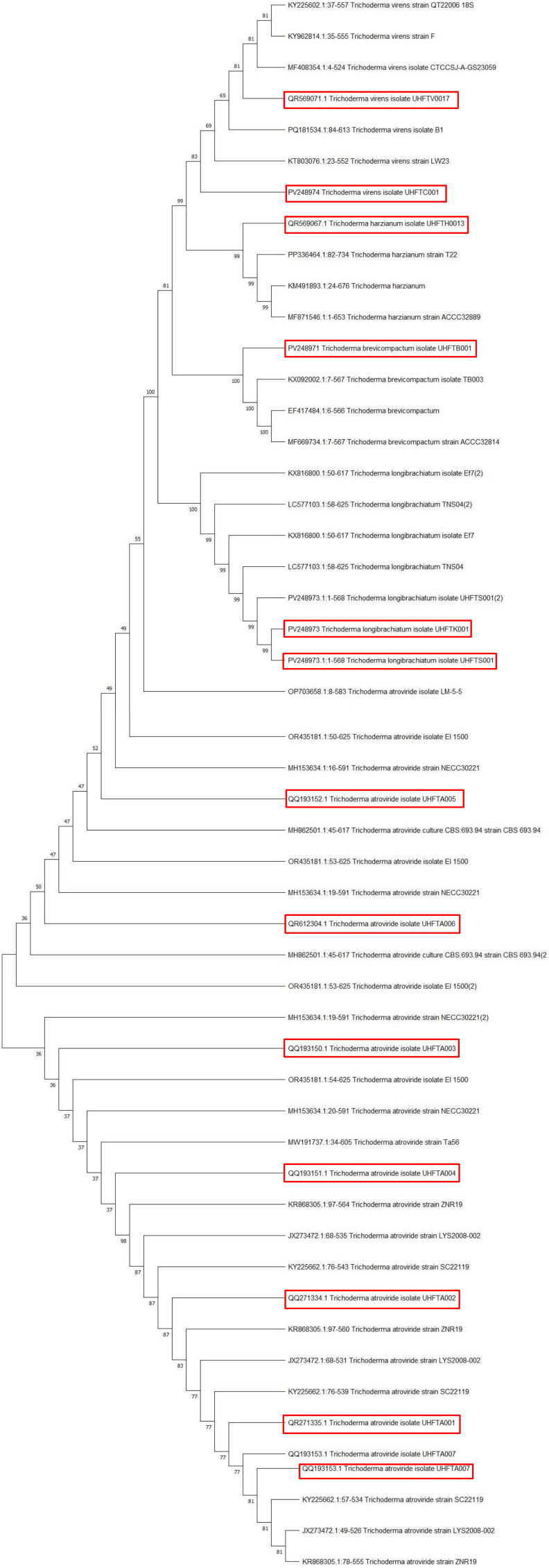
Phylogenic tree of isolates of *Trichoderma* species.

Different isolates of *Trichoderma* species were isolated and used for phylogenetic analysis (**Table 1**). The phylogenetic tree showed that *Trichoderma virens* UHFTV017 (QR569071.1) shared close affinity with the *T*. *virens* isolate CTCCSJ-A-CS23059 with 81% bootstrap support, *T*. *virens* UHFTC001 (PV248974) shared close affinity with the *T*. *virens* isolate LW23 with 69% bootstrap support, *Trichoderma harzianum* UHFTH0013 (QR569067.1) shared close affinity with the *T*. *harzianum* isolate T22 with 99% bootstrap support, *Trichoderma brevicompactum* UHFTB001 (PV248971) shared close affinity with the *T*. *brevicompactum* isolate TB003 with 100% bootstrap support, *Trichoderma longibrachiatum* UHFTS001 (PV254825) and *T*. *longibrachiatum* UHFTK001 (PV248973) shared close affinity with the *T*. *longibrachiatum* isolate Ef72 with 100% bootstrap support, *Trichoderma atroviride* UHFTA005 (QQ193152.1) shared close affinity with the *T*. *atroviride* isolate LM-5-5 with 100% bootstrap support, *T*. *atroviride* UHFTA006 (QR612304.1) shared close affinity with the *T*. *atroviride* isolate CBS693.94 with 52% bootstrap support, *T*. *atroviride* UHFTA003 (QQ193150.1) shared close affinity with the *T*. *atroviride* isolate CBS693.94 with 50% bootstrap support and the *T*. *atroviride* isolate CBS693.94 with 52% bootstrap support, *T*. *atroviride* UHFTA004 (QQ193151.1) shared close affinity with the *T*. *atroviride* isolate Ta56 with 37% bootstrap support, *T*. *atroviride* UHFTA002 (QQ271334.1) shared close affinity with the *T*. *atroviride* isolate ZNR19 with 83% bootstrap support, *T*. *atroviride* UHFTA001 (QR271335.1) shared close affinity with the *T*. *atroviride* isolate ZNR19 with 98% bootstrap support, and *T*. *atroviride* UHFTA007 (QQ193153.1) shared close affinity with the *T*. *atroviride* isolate ZNR19 with 81% bootstrap support (**Table 2**; **Figures 2**, **3**).

**Table 1:** List of *Trichoderma* species used.

Isolate	*Trichoderma* species
UHFTA001	*Trichoderma atroviride*
UHFTA002	*Trichoderma atroviride*
UHFTA003	*Trichoderma atroviride*
UHFTA004	*Trichoderma atroviride*
UHFTA005	*Trichoderma atroviride*
UHFTA006	*Trichoderma atroviride*
UHFTA007	*Trichoderma atroviride*
UHFTV017	*Trichoderma virens*
UHFTH0013	*Trichoderma harzianum*
UHFTB002	*Trichoderma brevicompactum*
UHFTS001	*Trichoderma longibrachiatum*
UHFTC001	*Trichoderma virens*
UHFTK001	*Trichoderma longibrachiatum*
UHFTB001	*Trichoderma brevicompactum*

**Table 2:** Molecular characterization of isolates of *Trichoderma* species.

Isolate	Location	Scientific name	Similarity (%)	Accession no.
UHFTA001	Nauni I	*Trichoderma atroviride*	100	QR271335.1
UHFTA002	Nauni I	*Trichoderma atroviride*	100	QQ271334.1
UHFTA003	Nauni II	*Trichoderma atroviride*	100	QQ193150.1
UHFTA004	Nauni II	*Trichoderma atroviride*	100	QQ193151.1
UHFTA005	Nauni III	*Trichoderma atroviride*	100	QQ193152.1
UHFTA006	Nauni III	*Trichoderma atroviride*	100	QR612304.1
UHFTA007	Nauni IV	*Trichoderma atroviride*	100	QQ193153.1
UHFTV017	Nauni IV	*Trichoderma virens*	100	QR569071.1
UHFTH0013	Nauni V	*Trichoderma harzianum*	100	QR569067.1
UHFTS001	Jubbal	*Trichoderma longibrachiatum*	100	PV254825
UHFTC001	Dharonda	*Trichoderma virens*	100	PV248974
UHFTK001	Jaterh	*Trichoderma longibrachiatum*	100	PV248973
UHFTB001	Ghumarwin	*Trichoderma brevicompactum*	99.82	PV248971

Garcia-Nunez et al. (2017) identified 10 isolates of *Trichoderma* with ITS-1 and ITS-4 of 600 bp amplicon size. Hermosa et al. (2000) identified several biocontrol isolates of *Trichoderma* of 500–600 bp amplicon size using ITS-1 and ITS-4 primers.

### 3.2 Qualitative screening of chitinase-producing strains

**Figure 4:**
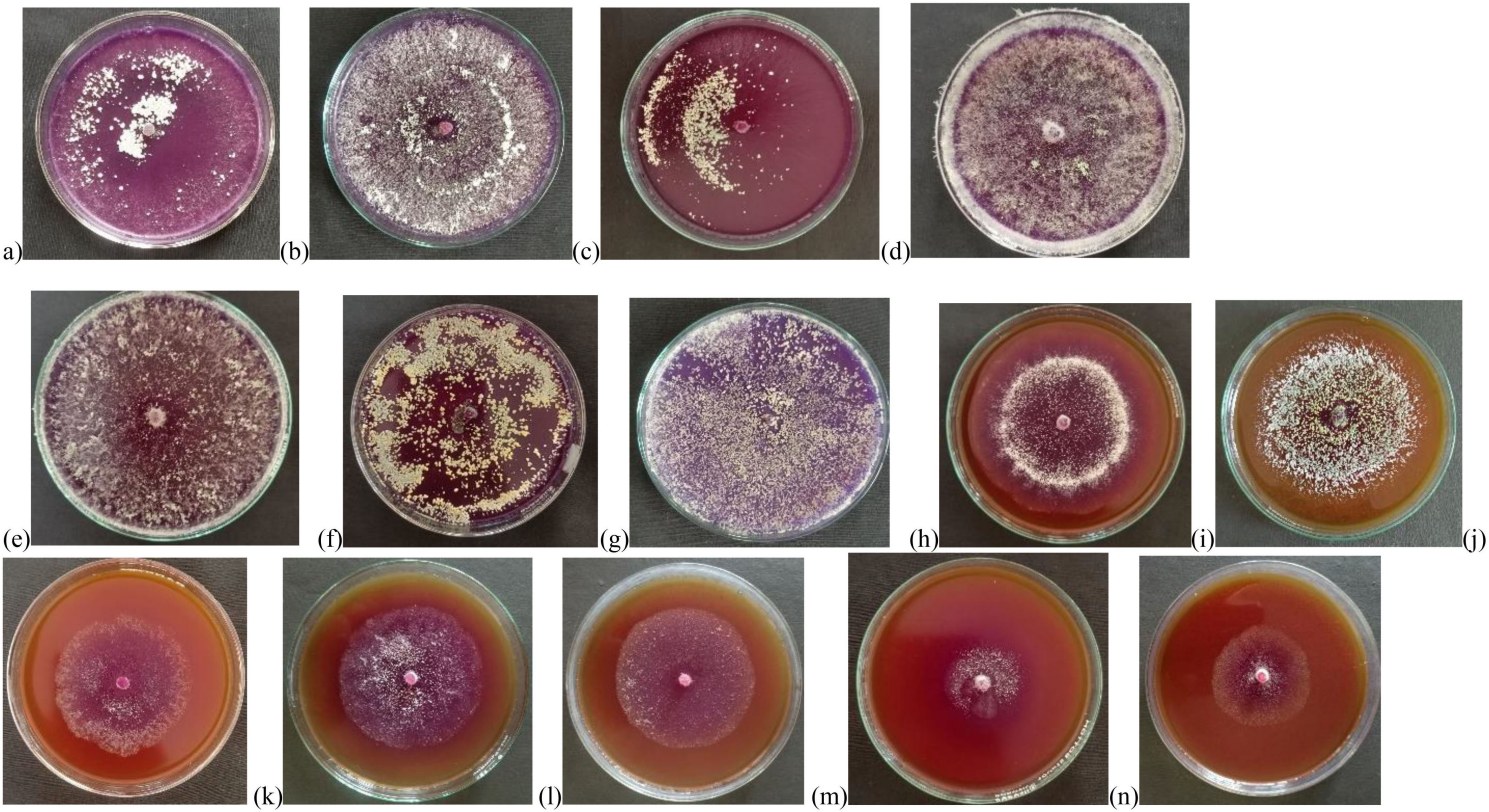
Qualitative analysis of chitinases producing isolates of Trichoderma spp. Where, **(a)** UHFTA005, **(b)** UHFTA006, **(c)** UHFTV017, **(d)** UHFTB002, **(e)** UHFTB001, **(f)** UHFTC001, **(g)** UHFTK001 (High chitinolytic activity), **(h)** UHFTA002, **(i)** UHFTA003, **(j)** UHFTA004, **(k)** UHFTA007, **(l)** UHFTS001 (Medium chitinolytic activity), **(m)** UHFTA001, **(n)** UHFTH0013 (Low chitinolytic activity).

The results of the qualitative screening for chitinase production showed that all isolates of *Trichoderma* spp. showed chitinolytic activity at varying extents. UHFTA005, UHFTA006, UHFTV017, UHFTB002, UHFTC001, UHFTK001, and UHFTB001 showed maximum chitinase activity; isolates UHFTA002, UHFTA003, UHFTA004, and UHFTA007 showed medium chitinase activity; and isolates UHFTA001 and UHFTH0013 showed minimum chitinase activity (**Table 3**; **Figure 4**).

**Table 3:** Qualitative assay of chitinolytic activity in *Trichoderma* isolates.

Trichoderma isolate	Diameter of the colored zone (mm)	Activity rate
UHFTA001	34e	Low
UHFTA002	52c	Medium
UHFTA003	70b	Medium
UHFTA004	55c	Medium
UHFTA005	90a	High
UHFTA006	90a	High
UHFTA007	52c	Medium
UHFTV017	90a	High
UHFTH0013	45d	Low
UHFTB002	90a	High
UHFTS001	55c	Medium
UHFTC001	90a	High
UHFTK001	90a	High
UHFTB001	90a	High
CD_(0.05)_	5.24	–

*Trichoderma* species produce the hydrolytic enzyme chitinase that is key to the hydrolysis of chitin, which is present in the cell wall of many phytopathogenic fungi. In the present study, the production of a hydrolytic zone around the colony indicated the breakdown of chitin by chitinases. The bromocresol purple dye resulted in a change in the pH toward alkalinity by changing the yellow color of the dye to purple. Different isolates of *Trichoderma* species showed significant differences in zone size, which is due to the varying capacities of the isolates to produce chitinase for the hydrolysis of chitin. Agrawal and Kotasthane (2012) reported that, out of 61 isolates of *Trichoderma* spp., 17 showed highest, 8 medium, and 12 low chitinase activity. Gonzalez et al. (2023) observed that *T. harzianum*, the *Trichoderma asperellum* isolate 12.2, and the *T*. *asperellum* isolate BP60 produced chitinase in a CCA medium by changing the color from orange to purple.

High activity (H), 76–100; moderate activity (M), 51–75; low activity (L), 25–50. Means in each column followed by the same lowercase letter(s) did not differ at CD_(0.05)_ according to Duncan’s multiple range test.

*CD_(0.05)_*, critical difference at 0.05 significance

High chitinolytic activity

Medium chitinolytic activity

Low chitinolytic activity

### 3.3 Optimization of the incubation period for chitinase production

**Figure 5:**
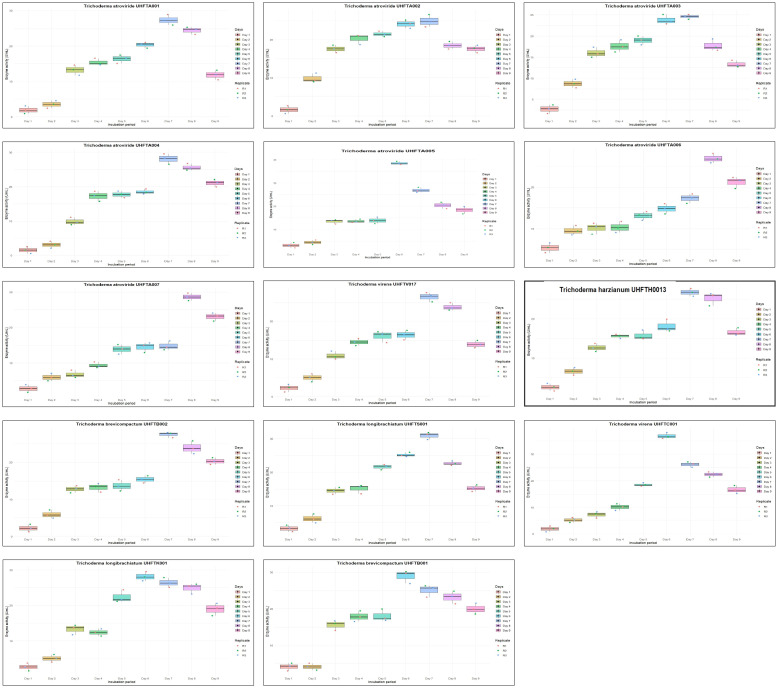
Box plot of optimization of incubation period for chitinase production.

Different isolates of *Trichoderma* species were evaluated for chitinase production under *in vitro* conditions, and chitinase activity was monitored regularly for 9 days at 30°C. **Table 4** illustrates the relationship between enzyme activity and incubation period, which revealed that the highest chitinase activity of the *T. atroviride* isolates UHFTA001, UHFTA002, UHFTA003, and UHFTA004; *T*. *harzianum* UHFTH0013; *T*. *virens* UHFTV017; *T*. *brevicompactum* UHFTB002; and *T*. *longibrachiatum* UHFTS001 peaked on day 7 of incubation, with values of 27.36, 24.88, 24.57, 28.13, 26.74, 26.43, 27.51, and 30.93 U/ml, respectively. However, in the case of *T*. *atroviride* UHFTA005, *T*. *virens* UHFTC001, *T*. *longibrachiatum* UHFTK001, and *T*. *brevicompactum* UHFTB001, the highest chitinase activity was achieved on day 6 of incubation, with activity values of 38.52, 36.82, 28.13, and 28.91 U/ml, respectively. In most of the isolates of *Trichoderma* species, the chitinase activity started increasing from the second day of incubation and continued its lag phase from the second day onward until day 7, where the chitinase activity reached its peak. With the increase in incubation period, the chitinase activity also increased, but declined beyond day 7 of incubation (**Figure 5**).

**Table 4:** Optimization of the incubation period for chitinase production.

Incubation period (days)	Enzyme activity (U/ml)													
	UHFTA001	UHFTA002	UHFTA003	UHFTA004	UHFTA005	UHFTA006	UHFTA007	UHFTV017	UHFTH0013	UHFTB002	UHFTS001	UHFTC001	UHFTK001	UHFTB001
1	1.93 ± 0.05	1.62 ± 0.01	2.71 ± 0.04	1.47 ± 0.01	3.17 ± 0.07	5.34 ± 0.10	3.79 ± 0.04	2.24 ± 0.04	2.55 ± 0.03	2.24 ± 0.006	3.17 ± 0.01	1.93 ± 0.04	2.55 ± 0.00	4.1 ± 0.08
2	3.48 ± 0.04	9.87 ± 0.23	8.75 ± 0.01	3.02 ± 0.006	4.41 ± 0.00	9.53 ± 0.11	5.96 ± 0.08	5.03 ± 0.04	6.58 ± 0.02	5.96 ± 0.11	6.14 ± 0.14	5.19 ± 0.04	5.03 ± 0.02	4.1 ± 0.04
3	13.25 ± 0.26	17.59 ± 0.01	16.04 ± 0.17	9.87 ± 0.13	13.41 ± 0.19	10.11 ± 0.24	6.78 ± 0.17	10.87 ± 0.14	12.67 ± 0.05	12.79 ± 0.06	14.49 ± 0.01	7.23 ± 0.16	13.25 ± 0.32	15.58 ± 0.27
4	15.42 ± 0.20	20.23 ± 0.36	17.59 ± 0.26	17.28 ± 0.27	13.56 ± 0.22	10.31 ± 0.20	9.37 ± 0.18	14.49 ± 0.04	15.58 ± 0.28	13.25 ± 0.16	15.11 ± 0.33	10.15 ± 0.18	13.41 ± 0.02	17.9 ± 0.27
5	16.35 ± 0.15	21.47 ± 0.16	18.99 ± 0.04	17.75 ± 0.02	13.87 ± 0.23	13.1 ± 0.07	13.87 ± 0.20	16.04 ± 0.37	15.73 ± 0.29	13.72 ± 0.28	21.62 ± 0.46	18.52 ± 0.39	22.4 ± 0.53	18.06 ± 0.45
6	20.34 ± 0.47	24.10 ± 0.07	23.79 ± 0.18	18.52 ± 0.42	38.52 ± 0.42	14.8 ± 0.13	14.49 ± 0.28	16.35 ± 0.16	18.06 ± 0.42	15.42 ± 0.009	25.19 ± 0.36	36.82 ± 0.17	28.13 ± 0.19	28.91 ± 0.51
7	27.36 ± 0.28	24.88 ± 0.37	24.57 ± 0.25	28.13 ± 0.30	26.89 ± 0.15	17.28 ± 0.09	14.8 ± 0.21	26.43 ± 0.17	26.74 ± 0.01	27.51 ± 0.24	30.93 ± 0.61	26.12 ± 0.09	26.43 ± 0.19	25.03 ± 0.45
8	24.41 ± 0.60	18.52 ± 0.03	17.75 ± 0.29	25.65 ± 0.56	20.38 ± 0.22	26.89 ± 0.09	28.6 ± 0.04	23.79 ± 0.58	25.19 ± 0.61	23.95 ± 0.43	22.65 ± 0.37	22.4 ± 0.04	24.88 ± 0.40	23.17 ± 0.43
9	11.86 ± 0.20	17.59 ± 0.06	13.35 ± 0.19	21.07 ± 0.06	18.33 ± 0.32	21.16 ± 0.28	23.02 ± 0.54	13.87 ± 0.04	15.58 ± 0.19	20.33 ± 0.04	15.22 ± 0.01	16.66 ± 0.34	18.98 ± 0.46	19.98 ± 0.29
CD_(0.05)_	2.16	1.94	1.93	1.76	1.97	1.69	1.70	2.01	2.03	2.00	1.49	2.04	2.47	2.41

Enzyme activity was assessed over a 9-day incubation period. Values are expressed as the mean ± standard error of three replicates, with critical difference (CD) at *p* > 0.05.

Optimization of the incubation period is a crucial factor for increasing the production of chitinase as it directly influences the secretion of enzymes and their metabolic activity. By optimizing the depletion of nutrients, instability of the enzymes and metabolic changes occur. Below the optimal incubation period, the activity decreases due to the slow binding of the substrate to the active site of the enzyme. At optimal incubation, the activity reaches its peak due to the presence of sufficient nutrients and the more efficient substrate binding to the enzyme’s active site. Other researchers have also optimized the incubation period for chitinase production across different microorganisms. El-Katatny et al. (2000) observed the maximum production of chitinase on day 7 of incubation in *T. harzianum*, while Gueye et al. (2020) observed a progressive increase of the chitinase activity of *T. asperellum* between 3 and 5 days, with the highest activity observed on day 5 of incubation with an enzyme activity of 2.81 U/ml. Brzezinska and Jankiewicz (2012) reported maximum chitinase production of the *Aspergillus niger* isolate LOCK 62 on day 6 of incubation. These findings provide evidence for the role of the incubation period in maximizing the enzymatic activity, with many microorganisms showing a peak in activity around days 6 and 7 of incubation.

### 3.4 Optimization of the substrate concentration for chitinase production

**Figure 6:**
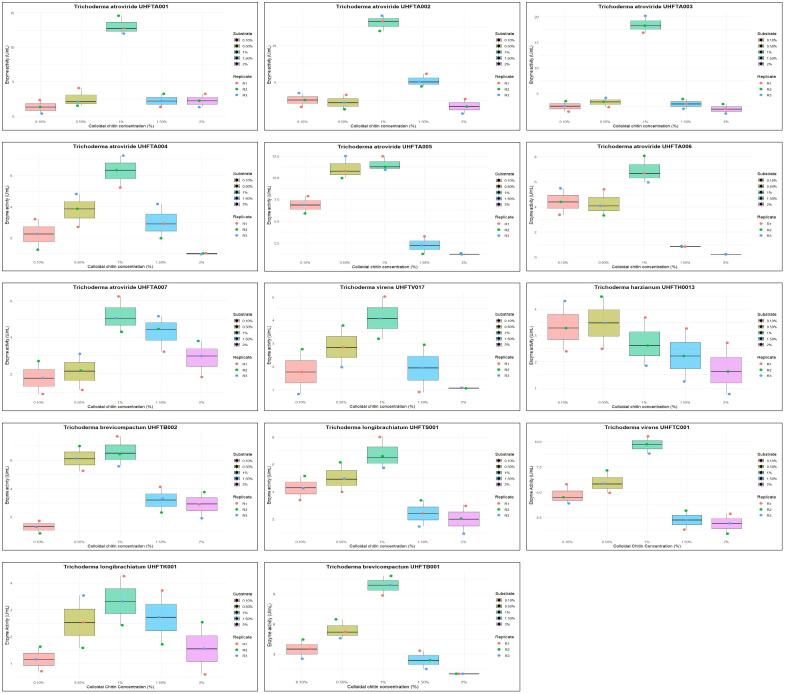
Box plot of optimization of substrate concentration for chitinase production.

The effect of the concentration of colloidal chitin (0.1%–2%) on enzyme production was examined to elucidate the best substrate concentration under controlled conditions of 30°C temperature and pH 5 over a period of 7 days. **Table 5** reveals that the highest chitinase activity in UHFTA001 (13.10 U/ml), UHFTA002 (13.10 U/ml), UHFTA003 (18.44 U/ml), UHFTA004 (6.27 U/ml), UHFTA005 (11.55 U/ml), UHFTA006 (6.89 U/ml), UHFTA007 (5.19 U/ml), UHFTV017 (4.10 U/ml), UHFTB002 (6.58 U/ml), UHFTS001 (6.74 U/ml), UHFTC001 (9.68 U/ml), UHFTK001 (3.33 U/ml), and UHFTB001 (9.84 U/ml) was observed at 1% concentration of colloidal chitin, indicating optimal chitinase production at this concentration, except for UHFTH0013 (3.48 U/ml) that had the highest activity at 0.5% concentration of colloidal chitin (**Figure 6**).

**Table 5:** Optimization of the substrate concentration for chitinase production.

Substrate (colloidal chitin, %)	Enzyme activity (U/ml)													
	UHFTA001	UHFTA002	UHFTA003	UHFTA004	UHFTA005	UHFTA006	UHFTA007	UHFTV017	UHFTH0013	UHFTB002	UHFTS001	UHFTC001	UHFTK001	UHFTB001
0.1	1.32 ± 0.01	2.55 ± 0.03	2.55 ± 0.01	2.24 ± 0.02	6.89 ± 0.01	4.41 ± 0.03	1.78 ± 0.04	1.78 ± 0.02	3.33 ± 0.03	1.31 ± 0.02	4.26 ± 0.07	4.26 ± 0.11	1.16 ± 0.02	3.48 ± 0.02
0.5	2.55 ± 0.27	2.24 ± 0.02	3.33 ± 0.07	3.79 ± 0.04	11.09 ± 0.22	4.26 ± 0.09	2.12 ± 0.01	2.86 ± 0.06	3.48 ± 0.00	6.12 ± 0.04	5.03 ± 0.08	5.98 ± 0.08	2.55 ± 0.01	5.41 ± 0.11
1	13.1 ± 0.24	13.10 ± 0.09	18.44 ± 0.38	6.27 ± 0.03	11.55 ± 0.19	6.89 ± 0.12	5.19 ± 0.07	4.10 ± 0.05	2.71 ± 0.06	6.58 ± 0.05	6.74 ± 0.15	9.68 ± 0.10	3.33 ± 0.04	9.84 ± 0.02
1.5	2.24 ± 0.04	5.19 ± 0.11	3.02 ± 0.02	3.02 ± 0.07	2.24 ± 0.01	0.85 ± 0.01	4.26 ± 0.09	1.93 ± 0.01	2.24 ± 0.01	3.17 ± 0.05	2.4 ± 0.03	1.90 ± 0.01	2.71 ± 0.003	2.40 ± 0.05
2	2.24 ± 0.02	1.70 ± 0.01	2.05 ± 0.04	1.00 ± 0.01	1.23 ± 0.02	0.22 ± 0.03	2.86 ± 0.05	1.08 ± 0.003	1.70 ± 0.03	2.89 ± 0.05	2.00 ± 0.01	2.24 ± 0.03	1.56 ± 0.006	1.01 ± 0.003
CD	1.98	1.73	1.94	1.57	1.49	1.73	1.72	1.59	1.93	1.67	1.80	1.93	1.79	1.35

Enzyme activity was assessed for different colloidal chitin concentrations. Values are expressed as the mean ± standard error of three replicates, with critical difference (CD) at *p* > 0.05.

Initially, more active sites of the enzymes are available for binding. Therefore, with the increase in substrate concentration, the reaction rate also increases due to more substrates binding to the active sites of the enzymes, leading to the release of more products. However, at a certain point, a further increase in the substrate concentration does not increase the reaction rate due to the saturation of all active sites of the enzymes, which prevents new substrates from binding, and due to the loss of enzyme activity. Optimal concentration of the substrate is a crucial factor for maximizing the production of chitinase and for increasing its biological efficacy against the chitin-exhibited phytopathogens. El-Katatny et al. (2000) concluded that the chitinase production of *T*. *harzianum* increased with an increase in the concentration of chitin up to 1%. These results are also in accordance with those of Elad et al. (1982), who concluded that the chitinase activity of *T*. *harzianum* increased up to a concentration of 1%.

### 3.5 Optimization of the pH for chitinase production

**Figure 7:**
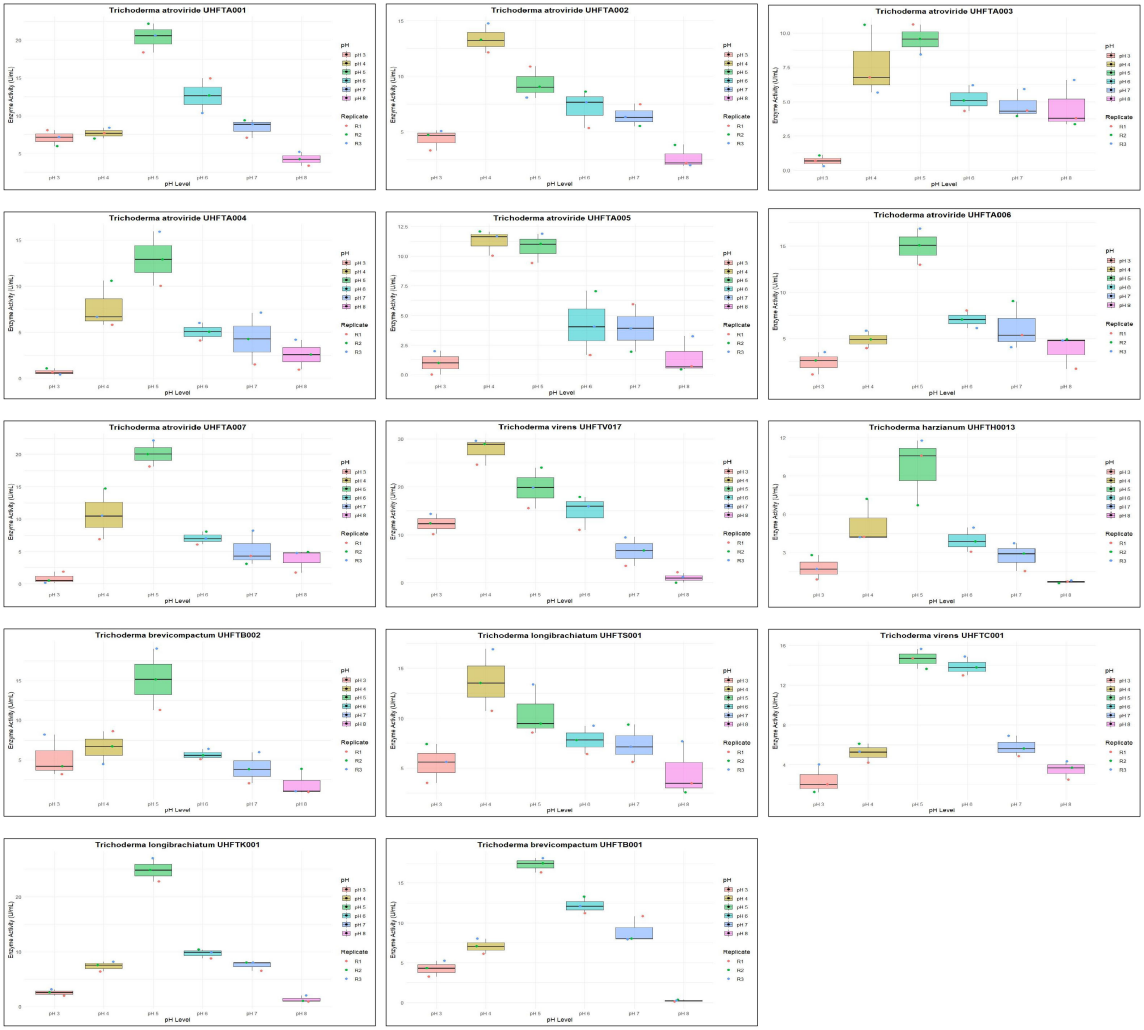
Box plot of optimization of pH for chitinase production.

All isolates of *Trichoderma* species were examined for the optimization of the pH to maximize chitinase production. The enzyme activity was estimated between pH 3 and pH 8 using colloidal chitin broth medium at 30°C, incubated for 7 days. As depicted in **Table 6**, there was a clear relationship between the pH of the medium and the activity of chitinase. The maximum production of chitinase of UHFTA001, UHFTA003, UHFTA004, UHFTA006, UHFTA007, UHFTH0013, UHFTB002, UHFTC001, UHFTK001, and UHFTB001 was recorded at pH 5, with enzymatic activity of 20.38, 9.53, 12.94, 14.96, 20.07, 9.68, 15.16, 14.65, 24.88, and 17.28 U/ml, respectively. On the other hand, the maximum production of chitinase of UHFTA002, UHFTA005, UHFTV017, and UHFTS001 was recorded at pH 4, with enzymatic activity of 13.35, 11.24, 27.67, and 13.72 U/ml, respectively (**Figure 7**).

**Table 6:** Optimization of the pH for chitinase production.

pH	Enzyme activity (U/ml)													
	UHFTA001	UHFTA002	UHFTA003	UHFTA004	UHFTA005	UHFTA006	UHFTA007	UHFTV017	UHFTH0013	UHFTB002	UHFTS001	UHFTC001	UHFTK001	UHFTB001
pH 3	7.05 ± 0.05	4.36 ± 0.54	0.69 ± 0.23	0.69 ± 0.20	1.00 ± 0.57	2.4 ± 0.7	0.85 ± 0.52	12.32 ± 1.21	1.78 ± 0.55	5.19 ± 1.52	5.5 ± 1.12	2.4 ± 0.30	2.55 ± 0.34	4.26 ± 0.56
pH 4	7.67 ± 0.40	13.3 ± 0.75	7.67 ± 1.49	7.67 ± 1.47	11.24 ± 1.74	4.88 ± 0.53	10.7 ± 2.25	27.67 ± 1.63	5.19 ± 1.00	6.58 ± 1.19	13.72 ± 1.79	5.19 ± 0.54	7.36 ± 0.52	7.05 ± 0.54
pH 5	20.3 ± 4.56	9.32 ± 0.83	9.53 ± 0.62	12.9 ± 1.69	10.77 ± 1.28	14.96 ± 2.85	20.07 ± 1.14	19.76 ± 2.44	9.68 ± 1.52	15.16 ± 2.22	10.46 ± 1.48	14.65 ± 0.97	24.88 ± 1.21	17.28 ± 0.52
pH 6	12.6 ± 1.32	7.22 ± 0.43	5.19 ± 0.54	5.03 ± 0.56	4.26 ± 1.55	7.05 ± 0.54	7.05 ± 0.56	14.96 ± 2.05	3.95 ± 0.55	5.65 ± 0.37	7.82 ± 0.80	13.87 ± 0.53	9.68 ± 0.48	12.17 ± 0.59
pH 7	8.44 ± 0.71	6.44 ± 0.58	4.72 ± 0.60	4.26 ± 1.61	3.93 ± 1.15	6.12 ± 1.49	5.19 ± 1.54	6.58 ± 1.75	2.71 ± 0.63	3.93 ± 1.13	7.36 ± 1.09	5.79 ± 0.58	7.51 ± 0.50	8.91 ± 0.94
pH 8	4.26 ± 0.51	2.66 ± 0.60	4.57 ± 1.00	2.55 ± 0.92	1.47 ± 0.89	3.79 ± 1.04	3.79 ± 1.04	1.00 ± 0.57	0.69 ± 0.06	1.93 ± 0.95	4.57 ± 1.57	3.49 ± 0.52	1.31 ± 0.34	0.23 ± 0.06
CD	2.89	2.45	2.88	2.90	3.03	2.81	3.80	3.86	2.75	4.06	2.97	1.29	1.24	1.96

Enzyme activity was assessed from pH 3 to pH 8. Values are expressed as the mean ± standard error of three replicates, with critical difference (CD) at *p* > 0.05.

Chitinase production was at its peak in the acidic medium, which declined under an alkaline environment, clearly suggesting the importance of the pH of the medium for maximum production of enzyme. During the exponential phase, the production of chitinase was the highest, enabling a more efficient breakdown of chitin. However, beyond this phase, the production entered a death phase, which was due to the exhaustion of all active sites of the enzyme. The denaturation of chitinase beyond the optimal pH reduced the enzyme’s efficiency. Therefore, maintaining an optimum pH is critical for its effective application in the management of targeted pathogens. Ekundayo et al. (2016) reported an optimum pH of 5 for the maximum chitinolytic activity of *Trichoderma viride*, while Sharma et al. (2021) reported the maximum production of chitinase by *T*. *harzianum* at pH 5.5.

### 3.6 Optimization of the temperature for chitinase production

**Figure 8:**
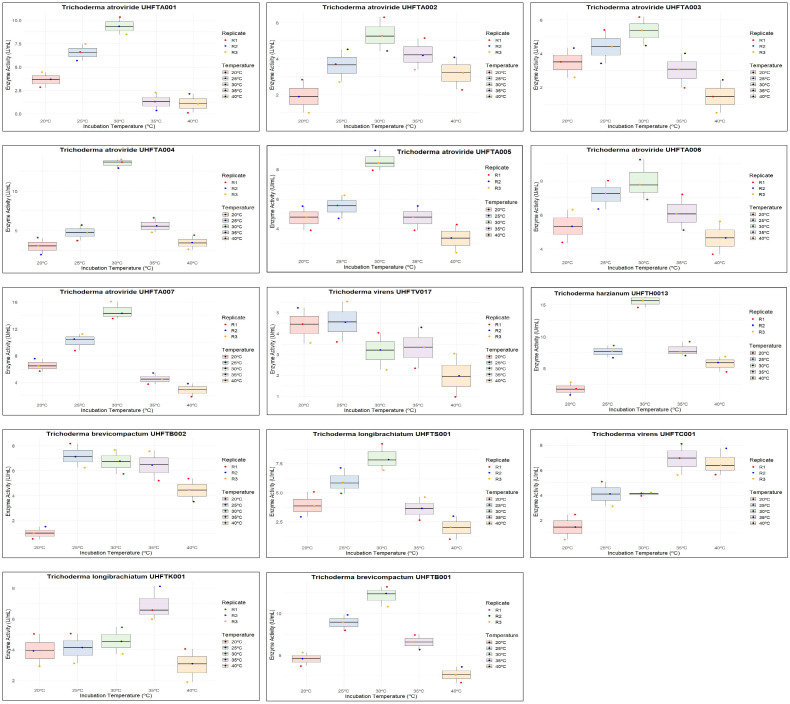
Box plot of optimization of incubation temperature for chitinase production.

To maximize the production of chitinase, an optimal temperature is a crucial factor. Different isolates of *Trichoderma* spp. were assessed for the optimization of the temperature between 20°C and 40°C. As shown in **Table 7**, the optimum temperature for chitinase production was at 30°C for most of the examined isolates of *Trichoderma* spp., including UHFTA001 (9.37 U/ml), UHFTA002 (5.34 U/ml), UHFTA003 (5.34 U/ml), UHFTA004 (13.56 U/ml), UHFTA005 (15.58 U/ml), UHFTA006 (7.98 U/ml), UHFTA007 (14.65 U/ml), UHFTH0013 (9.22 U/ml), UHFTS001 (7.98 U/ml), and UHFTB001 (12.12 U/ml). However, in UHFTV017 (4.57 U/ml) and UHFTB002 (7.20 U/ml), maximum chitinase activity was achieved when incubated at 25°C and 35°C in UHFTK001 (6.89 U/ml) and UHFTC001 (6.89 U/ml) (**Figure 8**).

**Table 7:** Optimization of the incubation temperature for chitinase production.

Temperature (°C)	Enzyme activity (U/ml)													
	UHFTA001	UHFTA002	UHFTA003	UHFTA004	UHFTA005	UHFTA006	UHFTA007	UHFTV017	UHFTH0013	UHFTB002	UHFTS001	UHFTC001	UHFTK001	UHFTB001
20	3.64 ± 0.09	1.92 ± 0.04	3.48 ± 0.07	3.02 ± 0.02	1.78 ± 0.01	5.34 ± 0.01	6.58 ± 0.06	4.41 ± 0.08	4.72 ± 0.11	1.00 ± 0.006	3.95 ± 0.05	6.58 ± 0.10	3.95 ± 0.03	4.55 ± 0.11
25	6.58 ± 0.05	3.64 ± 0.06	4.41 ± 0.006	4.72 ± 0.02	7.67 ± 0.03	7.20 ± 0.09	10.1 ± 0.18	4.57 ± 0.02	5.50 ± 0.13	6.43 ± 0.12	5.96 ± 0.08	6.89 ± 0.14	6.89 ± 0.16	8.90 ± 0.04
30	9.37 ± 0.04	5.34 ± 0.05	5.34 ± 0.09	13.5 ± 0.33	15.5 ± 0.03	7.98 ± 0.14	14.6 ± 0.22	3.17 ± 0.07	9.22 ± 0.14	7.20 ± 0.04	7.98 ± 0.11	4.10 ± 0.07	4.57 ± 0.07	12.1 ± 0.17
35	1.31 ± 0.03	4.26 ± 0.08	3.02 ± 0.03	5.65 ± 0.05	7.96 ± 0.17	6.12 ± 0.04	4.57 ± 0.10	3.33 ± 0.01	4.72 ± 0.10	6.74 ± 0.01	3.64 ± 0.01	4.10 ± 0.01	4.1 ± 0.02	6.55 ± 0.05
40	1.11 ± 0.02	3.21 ± 0.06	1.47 ± 0.01	3.45 ± 0.06	5.77 ± 0.10	4.65 ± 0.02	2.89 ± 0.03	1.99 ± 0.03	3.33 ± 0.03	4.44 ± 0.03	2.00 ± 0.01	1.45 ± 0.009	3.01 ± 0.05	2.65 ± 0.02
C.D.	1.83	1.60	1.87	1.73	2.08	2.03	1.25	1.80	1.60	1.93	2.20	1.59	1.07	2.00

Enzyme activity was assessed from 20°C to 40**°C** of incubation temperature. Values are expressed as the mean ± standard error of three replicates, with critical difference (CD) at *p* > 0.05.

The active site of an enzyme is a specific region where a substrate binds. When the temperature is below optimal for chitinase production by *Trichoderma* spp., the enzymatic activity is low due to the slower binding of the substrate; when the temperature is higher than optimal, denaturation of the enzyme occurs due to the distortion of its active site. Optimal temperature maximizes the enzyme activity due to the perfect shape of the active site, allowing enzyme to bind to the substrate in its maximum efficiency. Mohiddin et al. (2021) reported that *T. harzianum* showed the highest average chitinase activity at 30°C, except for the *T*. *harzianum* isolate BT3, which exhibited maximum production at 25°C. Mukhammadiev et al. (2020) reported that the highest activity (3.2 ± 0.08 U/ml) of *T*. *viride* was observed at 30°C compared with the other temperatures tested, and the enzyme synthesis declined at temperatures over 38°C. El-Katatny et al. (2000) reported that the optimum temperature for the production of chitinase by *T*. *harzianum* was around 30°C, with practically no growth being observed beyond 40°C.

### 3.7 Extraction, purification, and quantification of chitinase from *Trichoderma* species

Colloidal chitin broth medium was used for the extraction of chitinases from different isolates of *Trichoderma* spp. The supernatant collected from the medium was used as a crude enzyme extract, which was further purified with ammonium sulfate precipitation. The crude enzyme extract was suspended in 0%–45% ammonium sulfate, and the resulting precipitates were resuspended in citrate buffer and loaded into a Sephadex G-100 column for purification.

#### 3.7.1 Sodium dodecyl sulfate polyacrylamide gel electrophoresis

**Figure 9:**
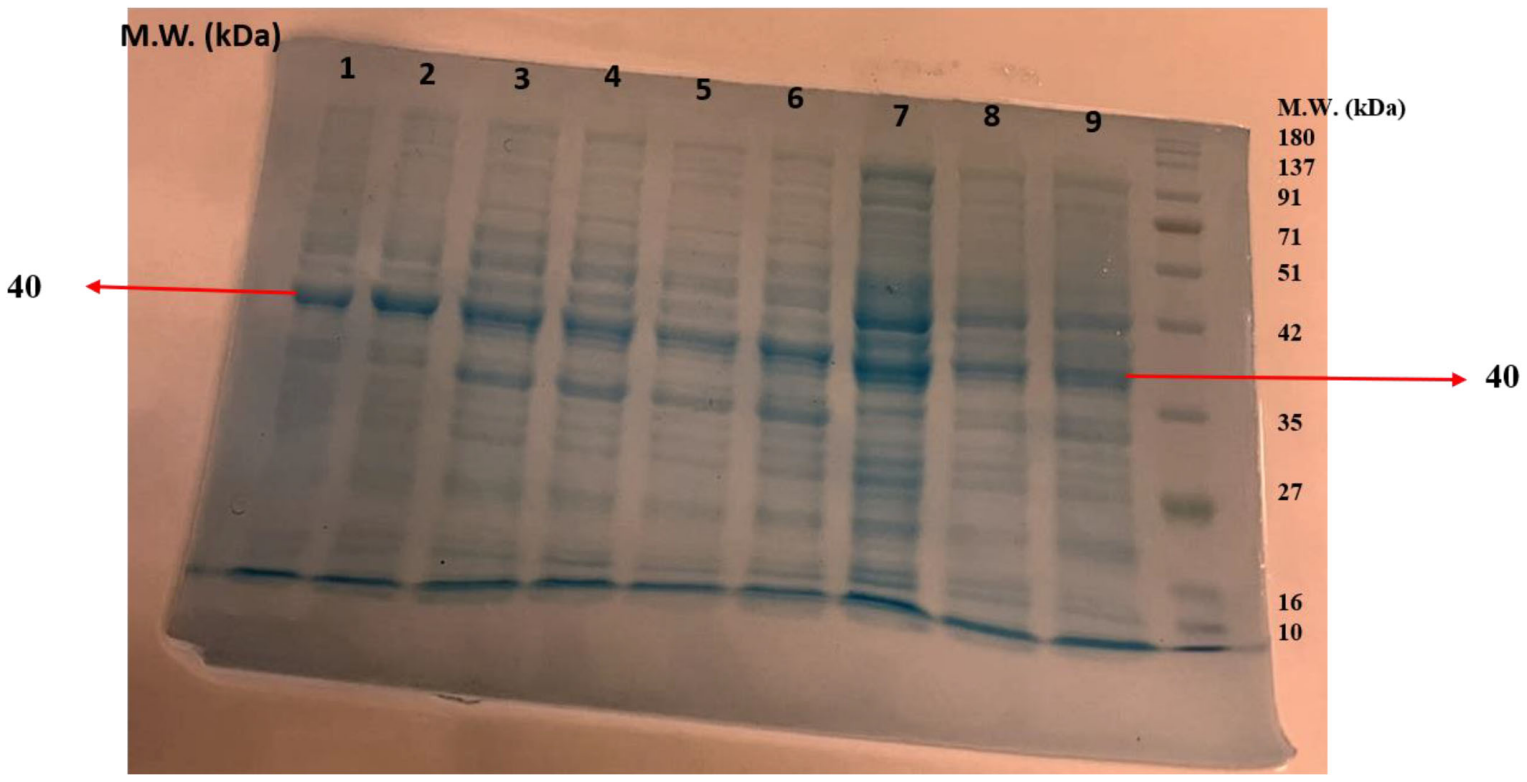
Molecular weight of chitinase (1: *T. atroviride* UHFTA006, 2: *T. virens* UHFTC001, 3: *T. brevicompactum* UHFTB001, 4: *T. virens* UHFTV017, 5: *T. atroviride* UHFTA002, 6: *T. longibrachiatum* UHFTK001, 7: *T. atroviride* UHFTA005, 8: *T. atroviride* UHFTA004, 9: *T. brevicompactum* UHFTB002).

SDS-PAGE was performed to determine the molecular weight and the purity of the enzyme. The partially purified protein from the ammonium sulfate precipitation was evaluated on 12% acrylamide gel. The presence of clear bands confirmed the purity of chitinase from the different species of *Trichoderma*. A protein molecular weight marker (10–180 kDa size) was used to determine the molecular weight of the protein. It was found that the molecular weight of the purified chitinases from the different species of *Trichoderma*, e.g., *T*. *atroviride* UHFTA006, *T*. *virens* UHFTC001, *T*. *brevicompactum* UHFTB001, *T*. *virens* UHFTV017, *T*. *atroviride* UHFTA002, *T*. *longibrachiatum* UHFTK001, *T*. *atroviride* UHFTA005, *T*. *atroviride* UHFTA004, and *T*. *brevicompactum* UHFTB002, was 40 kDa, which lies between 35 and 42 kDa of the molecular marker (**Figures 9**).

Purification is essential to remove contaminants and other associated proteins. The higher the purity of the enzyme, the higher the activity, stability, and efficiency against targeted fungal phytopathogens. A higher purity is also beneficial in the bioformulation of biofungicides to target specific pathogens. The molecular weight of an enzyme affects its stability in various growth conditions, its solubility, and its ability to bind to the substrate. The higher the molecular weight, the higher the efficiency of binding, whereas a lower molecular weight results in a fast diffusion rate. Harighi et al. (2007) recovered a 42-kDa chitinase (Chit42) from the supernatants of *T. atroviride*, and Kulkarni et al. (2010) also reported that the *Trichoderma* isolates Tv-10, Tv-21, Tv-23, and Th-13 exhibited 36- and 45-kDa bands in crude protein estimation using SDS-PAGE. Jenifer et al. (2014) also estimated a molecular mass of 46 kDa using SDS-PAGE.

#### 3.7.2 Quantification of the purified chitinases from the different isolates of *Trichoderma* species

Analysis of the data presented in **Table 8** highlighted that the highest specific activity of the purified chitinases was achieved in *T*. *atroviride* UHFTA005 (43.32 U/mg), followed by *T*. *atroviride* UHFTA006, with 38.20 U/mg specific activity. In contrast, the lowest specific activity was observed in the purified chitinase obtained from *T*. *atroviride* UHFTA001 (7.00 U/mg), followed by *T*. *harzianum* UHFTH0013 (7.10 U/mg), with 3.50 mg/ml protein concentration.

**Table 8:** Quantification of the purified chitinase from the different isolates of *Trichoderma* species.

Treatment	Chitinase activity (U/ml)	Specific activity (U/mg)
UHFTA001	27.30	7.00
UHFTA002	30.40	7.60
UHFTA003	24.85	7.10
UHFTA004	30.93	29.46
UHFTA005	36.82	43.32
UHFTA006	26.74	38.20
UHFTA007	28.13	29.92
UHFTV017	27.50	38.19
UHFTH0013	24.85	7.10
UHFTB002	26.90	30.92
UHFTS001	27.00	30.34
UHFTC001	26.00	32.50
UHFTK001	28.13	30.58
UHFTB001	28.60	30.75
CD_(0.05)_	1.09	1.07

*CD_(0.05)_*, critical difference at 0.05 significance

Specific activity is a key indicator in determining the purity and functionality of an enzyme. The higher the specific activity, the lower the non-enzymatic protein. A high specific activity value determines the higher purity of the protein, indicating that the total protein contains more active chitinase. This is a key parameter for the characterization of an enzyme, and it also represents the efficiency of the total protein present. Sayed et al. (2019) reported that, among all the tested species, *Trichoderma asperelloides* exhibited the highest chitinase activity (1.736 U/ml), with the highest total protein content (9.861 mg) and specific activity (0.176 U/mg). Similarly, Karaca and Eltem (2024) found that the chitinase enzyme activity of *Trichoderma* spp. ranged from 0.01 to 0.40 U/ml, with specific activity from 0.15 to 30.66 U/mg.

### 3.8 Effects of pH and temperature on the stability of chitinase

**Figure 10:**
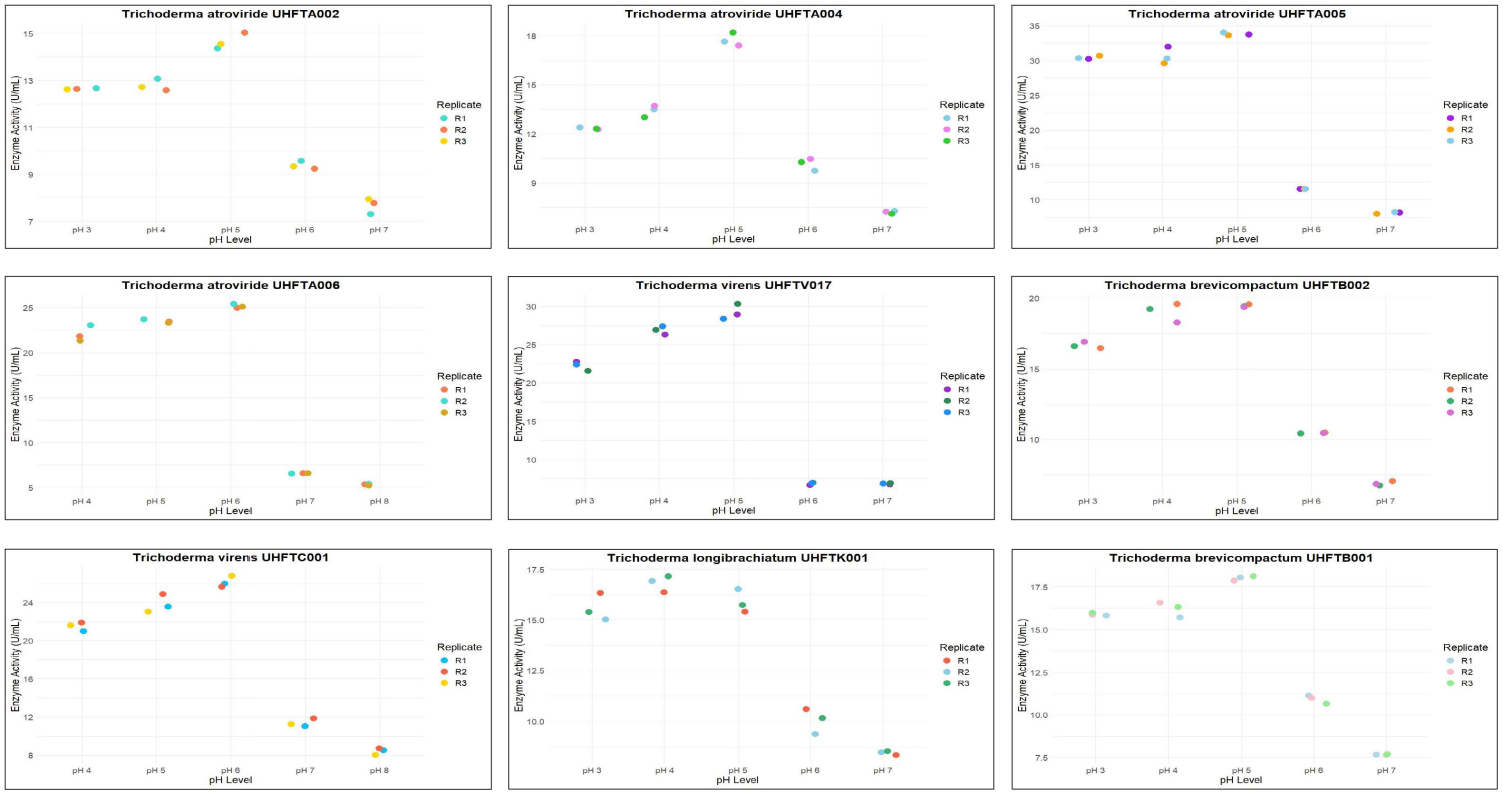
Dot plot of effect of pH on chitinase stability.

**Figure 11:**
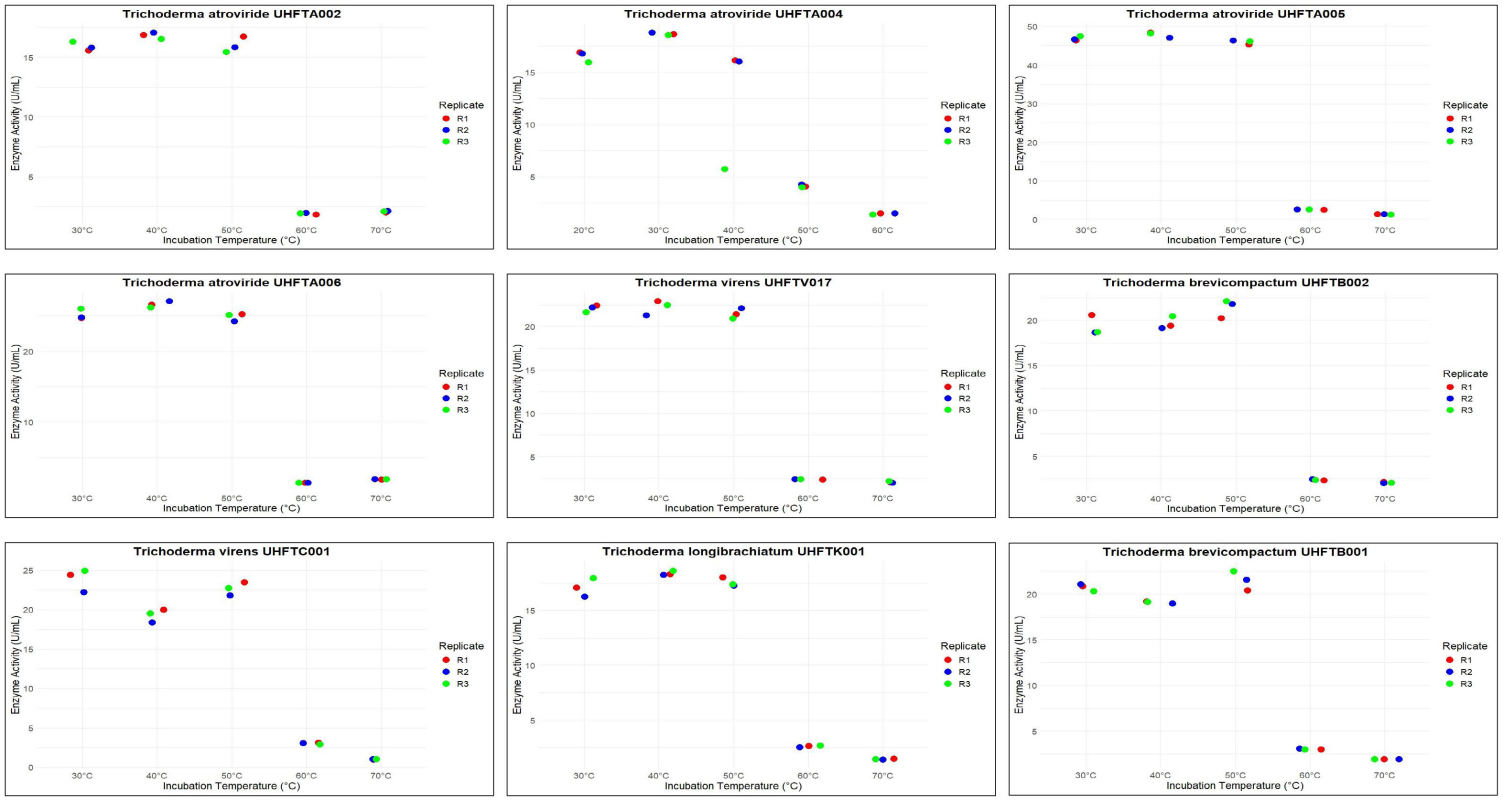
Dot plot of effect of temperature on chitinase stability.

Different isolates of *Trichoderma* spp. were selected on the basis of the highest specific activity of the purified chitinase and were assessed for stability at various pH levels (pH 3–7) and incubation temperatures (from 30°C to 70°C). The results demonstrated that all of the examined isolates of *Trichoderma* spp., including UHFTA002, UHFTA004, UHFTA005, UHFTA006, UHFTV017, UHFTB002, UHFTC001, UHFTK001, and UHFTB001, maintained high stability between a pH range of 3–7 and temperature of 30–50°C. A drastic fall in activity was observed beyond pH 5 and beyond 50°C. The stability of the chitinases from all isolates of *Trichoderma* spp. declined drastically, and a significant inactivation of the enzyme was observed at 70°C (**Tables 9**, **10** and **Figures 10**, **11**).

**Table 9:** Stability of the purified chitinase at different pH levels.

pH	Enzyme activity (U/ml)								
	UHFTA002	UHFTA004	UHFTA005	UHFTA006	UHFTV017	UHFTB002	UHFTC001	UHFTK001	UHFTB001
pH 3	12.63	12.32	30.42	22.09	22.48	16.67	21.48	15.58	15.89
pH 4	12.79	13.41	30.62	23.48	26.87	19.03	23.79	16.82	16.20
pH 5	14.65	17.75	33.80	25.19	29.22	19.46	26.12	15.89	18.03
pH 6	9.37	10.15	11.55	6.58	6.89	10.47	11.39	10.04	10.93
pH 7	7.67	7.20	8.13	5.34	6.89	6.90	8.44	8.44	7.64
CD_(0.05)_	0.53	0.63	1.09	0.67	1.18	0.49	1.07	1.07	0.49

One unit of chitinase activity is the amount of enzyme that produced 1 µmol min^−1^ ml^−1^ of *N*-acetyl-d glucosamine. Data represent the mean of three values at *p* < 0.05.

*CD_(0.05)_*, critical difference at 0.05 significance.

**Table 10:** Stability of the purified chitinase at different temperatures.

Temperature (°C)	Enzyme activity (U/ml)								
	UHFTA002	UHFTA004	UHFTA005	UHFTA006	UHFTV017	UHFTB002	UHFTC001	UHFTK001	UHFTB001
30	15.89	16.55	46.82	25.15	22.09	19.32	19.86	17.08	19.15
40	16.82	18.68	47.92	26.61	22.48	19.66	22.67	18.40	20.79
50	7.67	5.96	6.27	7.20	7.82	21.39	23.86	7.20	21.53
60	1.93	4.12	2.55	1.47	2.40	2.40	3.02	2.64	3.02
70	2.09	1.47	1.31	1.93	2.09	2.09	1.01	1.47	1.93
CD_(0.05)_	0.44	0.38	0.79	0.78	0.09	0.61	1.32	0.66	0.36

One unit of chitinase activity is the amount of enzyme that produced 1 µmol min^−1^ ml^−1^ of *N*-acetyl-d glucosamine. Data represent the mean of three values at *p* < 0.05.

*CD_(0.05)_*, critical difference at 0.05 significance.

The active site of an enzyme is highly sensitive to pH change, which affects the binding of the substrate and its catalytic function. A high pH can lead to denaturation due to the disruption of hydrogen bonding. Acidic pH can reduce the binding of the substrate to the active site of the enzyme due to the unfolding of protein. Microorganisms produce enzymes that must remain active in various environmental and soil conditions; therefore, it is important to determine the stability of the particular enzyme produced by a particular microorganism. In various bioformulations such as biopesticides and biofungicides, the activity of chitinase is crucial for maintaining its stability at a broad pH range.

The incubation temperature is an important factor that influences the stability of an enzyme, as high temperatures can disrupt the structure of the protein, leading to its denaturation. This suggests that the chitinases from the different species of *Trichoderma* are well adapted for functioning at medium to slightly high temperatures, i.e., 30–50°C, making them suitable for use in the biological control of various phytopathogens in the different agroclimatic zones of Himachal Pradesh. Jenifer et al. (2014) concluded that the chitinase from *T. viride* remained stable at pH 3–6 and up to 50°C. Ekundayo et al. (2016) also concluded that the optimal temperature for the chitinolytic activity of *T*. *viride* was 50°C and that it was stable at temperatures of 40–50°C and pH 6–7.

### 3.9 *In vitro* antifungal activity of chitinase against *Dematophora necatrix*

Different concentrations of the purified chitinases of 14 isolates of *Trichoderma* spp. were evaluated for their antifungal activity against *D. necatrix*. From **Table 11**, it is evident that, with the increase in the concentration of chitinase, the inhibition of *D. necatrix* also increased, with the highest inhibition observed in *T. atroviride* UHFTA005 at all concentrations, i.e., 0.15, 0.30, 0.45, and 0.60 μl with 55.55%, 64.44%, 77.77%, and 92.22% inhibition, respectively. However, minimum inhibition at 0.15 μl concentration was recorded in *T*. *atroviride* UHFTA001 (32.00%), and at 0.30 μl concentration, it was observed in *T*. *atroviride* UHFTA001 (57.77%). At 0.45 and 0.60 μl chitinase concentrations, minimum percent inhibition was recorded in *T*. *atroviride* UHFTA001, i.e., 68.88% and 83.33%, respectively, when compared with the control. All treatments were statistically significant.

**Table 11:** *In vitro* antifungal activity of chitinase against *Dematophora necatrix*.

Treatment	*D*. *necatrix* inhibition (%)			
	Chitinase concentration (μL)			
	0.15 μL	0.30 μL	0.45 μL	0.60 μL
UHFTA001	32.00h (34.64)	57.77d (49.08)	68.88h (56.01)	83.33g (65.78)
UHFTA002	41.11f (40.37)	61.11b (51.88)	74.44bcde (59.59)	87.77de (69.82)
UHFTA003	44.44e (41.79)	61.11b (51.60)	75.55abd (60.18)	87.77de (69.11)
UHFTA004	41.11f (39.57)	60.00bc (50.81)	74.44bcde (59.20)	87.77de (68.70)
UHFTA005	55.55a (48.38)	64.44a (53.96)	77.77a (62.69)	92.22a (74.18)
UHFTA006	55.55a (47.95)	64.44a (53.49)	77.77a (62.19)	91.11ab (72.95)
UHFTA007	38.88g (39.15)	60.00bc (50.28)	73.33cef (58.63)	86.66ef (68.28)
UHFTV017	53.33b (47.05)	63.33a (53.29)	76.66ab (61.87)	90.00bc (72.64)
UHFTH0013	38.88g (38.28)	57.77d (49.60)	71.11g (56.77)	85.55f (67.29)
UHFTB002	46.66d (43.06)	61.11b (51.46)	75.55abc (59.89)	88.88cd (71.39)
UHFTS001	38.88g (38.00)	58.88cd (49.94)	72.22fg (57.92)	85.55f (66.28)
UHFTC001	52.22b (46.76)	63.33a (52.44)	76.66ab (61.56)	90.00bc (71.64)
UHFTK001	52.22b (45.57)	63.33a (52.08)	76.66ab (60.95)	88.88cd (71.04)
UHFTB001	50.00c (44.98)	61.11b (51.05)	75.55abc (60.47)	88.88cd (70.75)
Control	0.00i (0.00)	0.00e (0.00)	0.00i (0.00)	0.00h (0.00)
CD_(0.05)_	1.07	1.03	1.30	1.68

Figures in parentheses are arcsine-transformed values.

*CD_(0.05)_*, critical difference at 0.05 significance.

Means followed by same lowercase letter(s) in a column do not differ significantly at the 5% level according to Duncan multiple range test.

### 3.10 *In vitro* antifungal activity of chitinase against *Sclerotium rolfsii*

The purified chitinases from the different isolates of *Trichoderma* spp. were assessed for their antifungal activity against *S. rolfsii*. As depicted in **Table 12**, the highest inhibition at 0.15 μl was recorded in *T. atroviride* UHFTA005 (22.22%), which was statistically at par with that in *T*. *atroviride* UHFTA006 (22.22%) and *T*. *virens* UHFTV017 (22.22%). Similarly, maximum inhibition at 0.30 μl was recorded in *T*. *atroviride* UHFTA005 (44.44%), which was statistically at par with that in *T*. *atroviride* UHFTA006 (44.44%) and *T*. *virens* UHFTV017 (44.44%). However, at 0.45 and 0.60 μl concentrations, maximum inhibition was observed in *T*. *atroviride* UHFTA005, with 68.88% and 91.11%, respectively. Minimum inhibition at 0.15 μl concentration was observed in *T*. *atroviride* UHFTA001 (3.33%) when compared with the control. All treatments were statistically significant.

**Table 12:** *In vitro* antifungal activity of chitinase against *Sclerotium rolfsii*.

Treatment	*S*. *rolfsii* inhibition (%)			
	Chitinase concentration (μL)			
	0.15 μL	0.30 μL	0.45 μL	0.60 μL
UHFTA001	3.33g (10.34)	30.00h (33.07)	44.44f (41.78)	74.44f (59.61)
UHFTA002	11.11f (19.61)	35.55e (37.16)	50.00e (45.57)	77.77e (62.04)
UHFTA003	16.66e(23.99)	38.88d (38.19)	52.22d (46.37)	78.88de (62.95)
UHFTA004	11.11f (19.58)	35.55e (36.16)	50.00e (44.68)	77.77e (61.76)
UHFTA005	22.22a (28.38)	44.44a (42.15)	68.88a (56.07)	91.11a (72.63)
UHFTA006	22.22a (28.11)	44.44a (41.83)	61.11b (51.63)	86.66b (69.17)
UHFTA007	11.11f (19.41)	33.33f (35.24)	48.88e (44.32)	77.77e (61.50)
UHFTV017	22.22a (27.86)	44.44a (41.38)	60.00b (50.51)	84.44c (67.28)
UHFTH0013	3.33g (10.66)	30.00h (33.31)	45.55f (42.43)	75.55f (60.34)
UHFTB002	16.66e (24.16)	38.88d (38.48)	54.44c (41.78)	80.00d (63.81)
UHFTS001	11.11f (19.22)	31.11g (33.88)	48.88e (43.94)	77.77e (61.09)
UHFTC001	21.11b (27.31)	42.22b (40.50)	55.55c (48.75)	83.33c (66.28)
UHFTK001	20.00c (26.55)	41.11c (39.86)	55.55c (48.16)	83.33c (65.89)
UHFTB001	17.77d (24.92)	38.88d (38.99)	55.55c (47.67)	83.33c (64.78)
Control	0.00h (0.00)	0.00i (0.00)	0.00g (0.00)	0.00g (0.00)
CD_(0.05)_	0.42	0.55	0.77	1.41

Figures in parentheses are arcsine-transformed values.

*CD_(0.05)_*, critical difference at 0.05 significance.

Means followed by same lowercase letter(s) in a column do not differ significantly at the 5% level according to Duncan multiple range test.

In the biological control of soilborne pathogens, namely, *D. necatrix* and *S. rolfsii*, through *Trichoderma* spp., chitinase plays a major role by degrading the chitin present in the cell wall of these fungi, thereby hydrolyzing chitin in the fungal cell wall, leading to the weakening of the cell structure, lysis of the cell, and, ultimately, death of the fungus. The degradation of chitin by chitinase also disrupts the integrity of fungal hyphae, making it more susceptible to antagonism by other microbes. In addition to suppressing the growth of fungal pathogens, chitinase plays an important role in the growth promotion of plants. Sharma et al. (2021) highlighted the potential inhibition of *D. necatrix* through chitinase from *Trichoderma* spp., while Gonzalez et al. (2023) highlighted that chitinase significantly inhibited the growth of *Fusarium oxysporum*.

### 3.11 Antifungal activity of chitinase against white root rot in pot culture conditions

From **Table 13**, it can be inferred that the chitinases from the different isolates of *Trichoderma* spp. not only reduced the incidence of white root rot but also promoted the health of plants in terms of plant height, stem girth, number of branches, and leaf area. Data pertaining to the effectiveness of *Trichoderma* spp. in **Table 13** revealed that minimum disease incidence (13.33%) was recorded in seedlings treated with 0.60 μl of *T*. *atroviride* UHFTA005 and *T*. *atroviride* UHFTA006, with 86.67% disease control compared with the control, thereby depicting maximum plant height, i.e., 89.73 and 87.70 cm, respectively. Similarly, maximum stem girth, leaf area, and number of branches were also recorded in both these treatments, i.e., 14.03 mm stem girth, 6.00 number of branches, and 15.87 cm^2^ leaf area in seedlings treated with *T*. *atroviride* UHFTA005, followed by *T*. *atroviride* UHFTA006 with 13.27 **mm** stem girth, 5.66 average number of branches, and 15.04 cm^2^ leaf area. However, minimum plant height, stem girth, number of branches, and leaf area were recorded in seedlings treated with 0.45 μl chitinase of *T*. *brevicompactum* UHFTB002, with respective values of 55.00 cm, 9.56 mm, 3.00, and 7.62 cm^2^. All treatments were statistically significant.

**Table 13:** Antifungal activity of chitinase against white root rot in pot culture conditions.

Treatment	Concentration (μl)	Plant height (cm)	Stem girth (mm)	No. of branches	Leaf area (cm^2^)	Disease incidence (%)	Disease control (%)
UHFTA005	0.45	60.10	10.03	3.00	9.55	53.33 (46.89)	46.67
UHFTA006	0.45	59.80	10.01	3.00	9.47	53.33 (46.89)	46.67
UHFTV017	0.45	57.63	9.89	3.00	9.45	60.00 (50.75)	40.00
UHFTC001	0.45	56.00	9.86	3.00	9.17	60.00 (50.75)	40.00
UHFTK001	0.45	55.50	9.76	3.00	9.06	60.00 (50.75)	40.00
UHFTB001	0.45	55.33	9.69	3.00	8.52	60.00 (50.75)	40.00
UHFTB002	0.45	55.00	9.56	3.00	7.62	66.67 (54.72)	33.33
UHFTA005	0.60	89.73	14.03	6.00	15.87	13.33 (21.40)	86.67
UHFTA006	0.60	87.70	13.27	5.66	15.04	13.33 (21.40)	86.67
UHFTV017	0.60	81.46	13.10	5.66	14.24	20.00 (26.55)	80.00
UHFTB002	0.60	81.46	12.84	5.66	13.71	20.00 (26.55)	80.00
UHFTC001	0.60	80.00	12.80	5.33	12.68	20.00 (26.55)	80.00
UHFTK001	0.60	77.60	12.71	5.33	12.01	26.67 (31.08)	73.33
UHFTB001	0.60	75.53	12.66	5.33	11.87	26.67 (31.07)	73.33
Control	–	35.56	7.59	1.00	5.53	100.00 (90.00)	0.00
CD_(0.05)_	**–**	1.79	0.48	0.18	0.50	1.18	**–**

Figures in parentheses are arcsine-transformed values.

*CD_(0.05)_*, critical difference at 0.05 significance.

Hood et al. (2023) demonstrated that *T. harzianum* β-1,4-glucosidase binds to and degrades the key structural polysaccharides of *M. phaseolina*—including α-1,3-glucan, β-1,3-glucan, β-1,3/1,4-glucan, and chitin—suggesting its role in the reduction of pathogen incidence by weakening the integrity of the fungal cell wall. Our research validated similar results, as the *Trichoderma* isolates producing chitinases significantly reduced the incidence of white root rot and enhanced plant health, highlighting chitinase as a crucial enzyme in the suppression of fungal pathogens. These findings underline the critical enzymatic involvement of *Trichoderma* in biocontrol.

### 3.12 Antifungal activity of chitinase against seedling blight in pot culture conditions

From **Table 14**, it can be inferred that the chitinases from the different isolates of *Trichoderma* spp. not only reduced the incidence of seedling blight but also promoted the health of plants in terms of plant height, stem girth, no of branches, and leaf area. Minimum disease incidence (26.67%) was recorded in seedlings treated with 0.60 μl of chitinase from *T*. *atroviride* UHFTA005, with 73.33% disease control, followed by *T*. *atroviride* UHFTA006, *T*. *virens* UHFTV017, *T*. *brevicompactum* UHFTB002, and *T*. *virens* UHFTC001, with 66.67% disease control in each case. Maximum disease incidence was recorded (86.67%) when seedlings were treated with 0.45 μl chitinase from *T*. *brevicompactum* UHFTB002. Data also revealed that maximum plant height (75.53 cm), stem girth (12.24 mm), number of branches (5.00), and leaf area (11.77 cm^2^) were recorded in seedlings treated with 0.60 μl chitinase from *T*. *atroviride* UHFTA005, followed by seedlings treated with 0.60 μl chitinase from *T*. *atroviride* UHFTA006, with 73.36 **cm** plant height, 12.14 mm stem girth, 5.00 average number of branches, and 11.75 cm^2^ leaf area. However, minimum plant height, stem girth, number of branches, and leaf area were recorded in seedlings treated with 0.45 μl chitinase from *T*. *brevicompactum* UHFTB002, with values of 42.26 cm, 8.26 mm, 2.00, and 5.80 cm^2^, respectively. All treatments were statistically significant.

**Table 14:** Antifungal activity of chitinase against seedling blight in pot culture conditions.

Treatment	Concentration (μl)	Plant height (cm)	Stem girth (mm)	No. of branches	Leaf area (cm^2^)	Disease incidence (%)	Disease control (%)
UHFTA005	0.45	53.76	9.43	2.66	7.20	66.67 (54.71)	33.33
UHFTA006	0.45	52.43	9.36	2.66	6.93	66.67 (54.72)	33.33
UHFTV017	0.45	51.03	9.22	2.66	6.73	73.33 (58.89)	26.67
UHFTC001	0.45	47.50	8.98	2.66	6.43	73.33 (58.88)	26.67
UHFTK001	0.45	46.46	8.81	2.66	6.03	80.00 (63.42)	20.00
UHFTB001	0.45	45.70	8.55	2.33	5.82	80.00 (63.41)	20.00
UHFTB002	0.45	42.26	8.26	2.00	5.80	86.67 (68.67)	13.33
UHFTA005	0.60	75.53	12.24	5.00	11.77	26.67 (31.07)	73.33
UHFTA006	0.60	73.36	12.14	5.00	11.75	33.33 (35.24)	66.67
UHFTV017	0.60	72.43	12.11	4.00	11.71	33.33 (35.24)	66.67
UHFTB002	0.60	72.03	11.73	4.00	11.59	33.33 (35.24)	66.67
UHFTC001	0.60	71.83	11.63	3.33	10.87	33.33 (35.24)	66.67
UHFTK001	0.60	69.00	11.37	3.33	10.71	40.00 (39.21)	60.00
UHFTB001	0.60	68.63	11.35	3.33	10.42	40.00 (39.21)	60.00
Control	–	41.33	7.86	1.66	5.65	100.00 (90.00)	0.00
CD_(0.05)_	**–**	1.98	0.43	0.13	0.36	1.72	**–**

Figures in parentheses are arcsine-transformed values. *CD_(0.05)_*, critical difference at 0.05 significance. Means followed by same lowercase letter(s) in a column do not differ significantly at the 5% level according to Duncan multiple range test.

The findings of Ribeiro dos Santos and Lima dos Santos (2025) underscored the multifaceted role of *Trichoderma* species in agriculture, emphasizing their capacity to colonize the rhizosphere, promote plant growth, and produce enzymes and secondary metabolites that contribute to pathogen suppression.

## 4 Conclusions

According to the results of this study, purified chitinase from the isolates of *Trichoderma* spp. can be successfully used not only to control soilborne pathogens of apple but also to promote plant health. The experimental results indicated that all of the isolates of *Trichoderma* spp. produced chitinases, but to varying extents. However, maximum production was observed when incubated for 7 days at pH 5 with 1% concentration of colloidal chitin at 30°C, whereas the enzyme was stable between pH 3 and 7 and temperature of 30–50°C. The isolates that showed the highest specific activity exhibited a molecular weight of 40 kDa. The chitinases from UHFTA005 and UHFTA006 exhibited the highest inhibition and disease control *in vitro* and in pot culture conditions. From all of the above, it can be concluded that the purified chitinases from isolates UHFTA005 and UHFTA006 can be effectively used against soilborne pathogens of apple.

## Data Availability

The original contributions presented in the study are included in the article/supplementary material. Further inquiries can be directed to the corresponding author.
